# The genome structure of *Arachis hypogaea* (Linnaeus, 1753) and an induced *Arachis* allotetraploid revealed by molecular cytogenetics

**DOI:** 10.3897/CompCytogen.v12i1.20334

**Published:** 2018-03-14

**Authors:** Eliza F. de M. B. do Nascimento, Bruna V. dos Santos, Lara O. C. Marques, Patricia M. Guimarães, Ana C. M. Brasileiro, Soraya C. M. Leal-Bertioli, David J. Bertioli, Ana C. G. Araujo

**Affiliations:** 1 University of Brasilia, Institute of Biological Sciences, Campus Darcy Ribeiro, CEP 70.910-900, Brasília, DF, Brazil; 2 Embrapa Genetic Resources and Biotechnology, PqEB W5 Norte Final, CP 02372, CEP 70.770-917, Brasília, DF, Brazil; 3 Catholic University of Brasilia, Campus I, CEP 71966-700, Brasília, DF, Brazil; 4 Center for Applied Genetic Technologies, University of Georgia, 111 Riverbend Road, 30602-6810, Athens, Georgia, USA

**Keywords:** Chromosomes, DNA content, FISH, GISH, heterochromatic bands, LTR-retrotransposons, peanut, rDNA

## Abstract

Peanut, *Arachis hypogaea* (Linnaeus, 1753) is an allotetraploid cultivated plant with two subgenomes derived from the hybridization between two diploid wild species, *A. duranensis* (Krapovickas & W. C. Gregory, 1994) and *A. ipaensis* (Krapovickas & W. C. Gregory, 1994), followed by spontaneous chromosomal duplication. To understand genome changes following polyploidy, the chromosomes of *A. hypogaea*, IpaDur1, an induced allotetraploid (*A. ipaensis* × *A. duranensis*)^4x^ and the diploid progenitor species were cytogenetically compared. The karyotypes of the allotetraploids share the number and general morphology of chromosomes; DAPI^+^ bands pattern and number of 5S rDNA loci. However, one 5S rDNA locus presents a heteromorphic FISH signal in both allotetraploids, relative to corresponding progenitor. Whilst for *A. hypogaea* the number of 45S rDNA loci was equivalent to the sum of those present in the diploid species, in IpaDur1, two loci have not been detected. Overall distribution of repetitive DNA sequences was similar in both allotetraploids, although *A. hypogaea* had additional CMA_3_^+^ bands and few slight differences in the LTR-retrotransposons distribution compared to IpaDur1. GISH showed that the chromosomes of both allotetraploids had preferential hybridization to their corresponding diploid genomes. Nevertheless, at least one pair of IpaDur1 chromosomes had a clear mosaic hybridization pattern indicating recombination between the subgenomes, clear evidence that the genome of IpaDur1 shows some instability comparing to the genome of *A. hypogaea* that shows no mosaic of subgenomes, although both allotetraploids derive from the same progenitor species. For some reasons, the chromosome structure of *A. hypogaea* is inherently more stable, or, it has been at least, partially stabilized through genetic changes and selection.

## Introduction

The genus *Arachis* (Linnaeus, 1753) is native to South America, with *Arachis* as the largest botanical section. Most species in this section are diploids (2n = 2x = 20), but there are a few aneuploids and two tetraploids: *A. hypogaea* (Linnaeus, 1753), the cultivated peanut (groundnut) and *A. monticola* (Krapovickas & Rigoni, 1958) (2n = 4x = 40) (Krapovickas and Gregory 1994, Valls and Simpson 2005). *A. hypogaea* has its origin estimated between 3,500 and 9,400 years ago (Bonavia 1982, Simpson et al. 2001, Bertioli et al. 2016), from one or few events of hybridization between two wild diploid species, followed by spontaneous polyploidization (Singh 1986, Kochert et al. 1996, Grabiele et al. 2012).

Whereas the chromosomes of *A. hypogaea* are of mostly similar size and metacentric, cytogenetic analysis can distinguish two different genome components: the A subgenome comprising ten pairs of chromosomes, with the centromeres strongly stained by DAPI, including the small pair termed ‘A’ (Husted 1936) and the B subgenome, with another ten pairs of chromosomes that have no, or just weak DAPI^+^ bands (Seijo et al. 2004, 2007, Robledo and Seijo 2010). Fluorescence in situ hybridization (FISH) and many different lines of evidence show that the distribution of rDNA loci and heterochromatic DNA inww *A. hypogaea* are almost equivalent to the sum of those of the progenitor diploid species: *A. duranensis* (Krapovickas & W. C. Gregory, 1994), which has A genome chromosome, and *A. ipaensis* (Krapovickas et W. C. Gregory, 1994), which has B genome chromosomes (Grabiele et al. 2012, Robledo et al. 2009, Robledo and Seijo 2010). The only exception to this is that in both diploid species, the 45S rDNA hybridization signals bear the thread-like constriction of the pair of chromosomes SAT that strongly suggests transcriptional activity (Fernández and Krapovickas 1994). However, in *A. hypogaea*, the secondary constrictions observed on the B subgenome chromosomes have been silenced (Seijo et al. 2004), a common event in polyploids called nucleolar dominance (Navashin 1934, Preuss and Pikaard 2007).

Dhillon et al. (1980), using renaturation kinetics, estimated that 64 % of the *A. hypogaea* genome was composed of repetitive sequences. Genomic in situ hybridization (GISH) on chromosomes of *A. hypogaea*, with labeled whole genomic DNA from *A. duranensis* and *A. ipaensis* hybridized concomitantly showed that whilst the probes hybridize indistinctly to some genomic regions, the chromosomes of A and B genome components (A and B subgenomes) are easily distinguishable (Seijo et al. 2007). Since the hybridization kinetics favors repetitive DNA sequences, this indicates that whereas the *A. hypogaea* A and B subgenomes share common repetitive DNA sequences with both diploid progenitors, in other aspects, the repetitive sequences are quite distinct between the subgenomes (Raina and Mukai 1999, Seijo et al. 2007).

Cytogenetic analysis mainly reveals the faster evolving repetitive DNA sequences; therefore, it tends to emphasize the differences between the subgenomes in allopolyploids. On the other hand, observations using genetic mapping and genes in *Arachis* tended to detect the similarities between the subgenomes: high collinearity between A and B subgenomes has been shown by comparing genetic linkage maps and sequencing of homeologous regions (Burow et al. 2001, Shirasawa et al. 2013, Bertioli et al. 2013, Bertioli et al. 2016). In addition, sequencing has shown very high DNA identity between A and B genes: around 97 % (Ramos et al. 2006, Nielen et al. 2012, Moretzsohn et al. 2013). The distinct fractions are thought to have evolved independently, following the evolutionary divergence of the progenitor species, which is estimated to have occurred 2–3 million years ago (Nielen et al. 2010, Bertioli et al. 2013, Moretzsohn et al. 2013, Samoluk et al. 2015a, Bertioli et al. 2016).

An important step in the understanding the genetics of many crops has been obtained by whole genome sequencing. However, for *A. hypogaea*, the very high similarity of the subgenomes makes the characterization of its genome, at the whole genome level, very challenging, although various lines of evidence suggested that the progenitor genomes had undergone relatively few changes since polyploidization (Fávero et al. 2015, Foncèka et al. 2012, Shirasawa et al. 2012, Bertioli et al. 2016, Chen et al. 2016). Phenotypic and genetic observations of progeny derived from crosses between *A. hypogaea* and the induced allotetraploid [(*A. ipaensis* K30076 × *A. duranensis* V14167)^4x^] (Fávero et al. 2006), here called IpaDur1, strongly supported the close relationship between the diploid genomes and corresponding *A. hypogaea* subgenomes (Foncèka et al. 2012, Shirasawa et al. 2012).

The availability of the genome sequences of two representatives of *A. hypogaea* diploid progenitor species, *A. duranensis* V14167 and *A. ipaensis* K30076, (Bertioli et al. 2016) made possible to analyze their assembled chromosomal pseudomolecules. Homeologous chromosomes were given corresponding numbers based on previous genetic linkage maps, which most unfortunately, do not have correspondence with cytogenetic chromosome assignments (Bertioli et al. 2016). Comparisons of the diploid genome sequences with those of *A. hypogaea* confirmed the high sequence identity between the diploid genomes and their corresponding tetraploid components (Bertioli et al. 2016, Chen et al. 2016). However, as may have been expected for closely related highly collinear homeologous chromosomes, some recombination between the subgenomes of *A. hypogaea* was detected. Small terminal chromosome regions have changed from the expected genome formula of AABB, to AAAA, and others had changed to BBBB. These events were similar to, but smaller than, the recombination between subgenomes previously detected using genetic markers in this same induced allotetraploid IpaDur1 (Leal-Bertioli et al. 2015). There were also, in *A. hypogaea*, distinct signs of migration of B subgenome alleles to A subgenome, especially in collinear homeologs (Bertioli et al. 2016).

In addition to genetic recombination between *A. hypogaea* subgenomes, other genomic changes are likely to have occurred following what McClintock (1984) termed as “genomic shock” of polyploid formation (Adams and Wendel 2005). Such changes may be caused by transposable element activation and re-organization of repetitive DNA sequences. While the overall patterns of GISH and evidences of the abundance of retrotransposons (Nielen et al. 2010, 2012, Samoluk et al. 2015a) indicate that, there has not been a mass movement of transposons between the genomes, or large-scale re-organization of repetitive DNA sequences, further investigations using IpaDur1 could disclose modifications that are frequently found in new hybrids (Comai 2000, Kashkush et al. 2003, Shcherban 2013, Kim 2017).

Interestingly, the recent cytogenetic observations of Zhang et al. (2016) showed that whilst the subgenome B chromosomes of *A. hypogaea* were very similar compared to its *A. ipaensis* counterpart, there were differences between the A subgenome and *A. duranensis* chromosomes. The authors suggested the participation of distinct *A. duranensis* accessions in the origin of *A. hypogaea*. However, this is not consistent with DNA marker data, which strongly implies a single origin (Kochert et al. 1991, 1996, Grabiele et al. 2012, Moretzsohn et al. 2013). Instability in the A subgenome chromosomes since polyploidy is an alternative explanation for that.

With the aim of understanding genome changes that have occurred after the polyploidization in *A. hypogaea*, a detailed comparative cytogenetic study of *A. hypogaea*, IpaDur1and progenitor diploid species is here presented. It was expected that the recently synthesized allotetraploid would undergo similar changes to those in *A. hypogaea* in the first years following polyploidization. Here is shown that IpaDur1 shows some alterations also observed in *A. hypogaea*, such as possible A genome nucleolar dominance, genome deletions and transposons activity. However, further alterations in IpaDur1, such as the smaller number of 45S rDNA loci and evident large-scale recombination between subgenomes in at least one chromosome pair of IpaDur1 were here evidenced. Current data contributes directly to the understanding of immediate effects of allotetraploidization in *Arachis* and to the overall understanding of *Arachis* genomes.

## Material and methods

### Plant material

Seeds from the wild diploid species (2n = 20) *A. duranensis*, accession V14167 and *A. ipaensis*, accession K30076; the allotetraploids (2n = 40) A. hypogaea subsp. fastigiata var. fastigiata ‘IAC Tatu-ST’ (AABB) and the induced allotetraploid IpaDur1 (*A. ipaensis* K30076 × *A. duranensis* V14167)^4x^ (Fávero et al. 2006) (BBAA) were obtained from the Embrapa Genetic Resources and Biotechnology Active Germplasm Bank (genotypes summarized in Table 1), and growing plants were maintained in open plan greenhouse.

**Table 1.** DNA content and size, CMA_3_^+^ bands and distribution of the in situ hybridization signals (GISH and FISH) on chromosomes of the four *Arachis* genotypes.

	Genotypes	*A. duranensis*	*A. ipaensis*	IpaDur1	*A. hypogaea*
	**Karyotype formula**	9 m + 1 sm	9 m + 1 sm	18 m + 2 sm	18 m + 2 sm
	**DNA content (2C) (pg)**	2.62	3.34	5.92	5.70
	**Size (1C) (Gb**	1.28	1.63	2.89	2.79
	**CV (%)**	2.67	4.14	2.36	3.25
	**CMA**_3_ **^+^**	Proximal regions on cyt-A10*	Proximal region on cyt-B10*	Proximal region on cyt-A10* and cyt-B10	Proximal regions on cyt-A10*, cyt-B10 and another three pairs
**GISH (genomic probes)**	**IpaDur1**	–	–	On all chromosomes, for both subgenomes. Few signals on centromeres of A subgenome chromosomes and terminal regions. Cyt-B10 entirely covered by signals	On all chromosomes, for both subgenomes. Seldom signals on cyt-A9. Few signals on centromeres of A subgenome chromosomes and terminal regions. Cyt-B10 entirely covered by signals
	***A. hypogaea***	–	–	On all chromosomes, for both subgenomes. Seldom signals on cyt-A9. Few signals on centromeres of A subgenome chromosomes and terminal regions. Cyt-B10 with alternated pattern	On all chromosomes, of both subgenomes. Seldom signals on cyt-A9. Few signals on centromeres of A subgenome chromosomes and terminal regions. Cyt-B10 entirely covered by signals
	***A. duranensis* and *A. ipaensis***	–	–	Higher affinity to chromosomes of each corresponding subgenome. Hybridized poorly on cyt-A9, centromeres of A chromosomes and terminal regions of all chromosomes. Cyt-B10 with mosaic pattern	Higher affinity to chromosomes of each corresponding subgenome. Hybridized poorly on cyt-A9, centromeres of A chromosomes and terminal regions of all chromosomes. Cyt-B10 with higher affinity to *A. ipaensis* probe
**rDNA FISH**	**5S**	Proximal region on cyt-A3	Proximal region on cyt-B3	Interstitial region on cyt-A3 and proximal region on cyt-B3	Interstitial region on cyt-A3 and proximal region on cyt-B3
	**45S**	Proximal region on cyt-A2 and A10*	Proximal region on cyt-B3 and B10* and on terminal region on cyt-B7	Proximal region on cyt-A2; A10* and B10	Proximal regions on cyt-A2; A10*; B3 and B10 and in terminal regions on cyt-B7
**LTR-RT FISH**	**RE128-84**	Dispersed on arms and proximal regions of all chromosomes. Seldom detected on centromeric and terminal regions	Dispersed on the arms and proximal regions of most chromosomes. Lacking on two pairs. Seldom detected on centromeric and terminal regions	Dispersed on the arms and proximal regions of most chromosomes. Lacking on one pair of chromosome of the subgenome A Seldom detected on centromeric and terminal regions	Dispersed on arms and proximal regions of most chromosomes. Lacking on cyt-A9 and cyt-A10. Seldom detected on centromeric and terminal regions
**LTR-RT FISH**	**Pipoka**	Dispersed on arms and proximal regions of most chromosomes. Poorly on cyt-A9 and cyt-A10. Seldom detected on centromeric and terminal regions	Dispersed on the arms and proximal regions of most chromosomes. Seldom detected on centromeric and terminal regions	Dispersed on the arms and proximal regions of most chromosomes. Lacking on cyt-A9, cyt-A10 and on two pairs of A subgenome. Seldom detected on centromeric and terminal regions	Dispersed on the arms and proximal regions of few chromosomes. Lacked on cyt-A9, cyt-A10. Seldom detected on centromeric and terminal regions
	**Athena**	Dispersed on arms and proximal regions of most chromosomes. Seldom on centromeric and terminal regions	Dispersed on the arms and proximal regions of most chromosomes Lacking on terminal regions of all chromosomes	Dispersed on the arms and proximal regions of most chromosomes on B subgenome. Lacking on cyt-A9 and cyt-A10. Seldom detected on centromeric and terminal regions	Dispersed on the arms and proximal regions of most chromosomes, Lacking on cyt-A9 and cyt-A10. Seldom detected on centromeric and terminal regions

CV: coefficient of variance; m: metacentric; sm: submetacentric; *: NOR (Nucleolar Organizing Region); –: not analysed.

### Genome sizes

Genome sizes were estimated using the CyFlow Space system (Sysmex Partec GmbH, Görlitz, Germany), with leaf cells labeled with propidium iodide, as described by Galbraith et al. (1983). Leaflets of the third leaf, from three weeks old plants were removed from five different individuals, for each genotype. Samples were distributed as three technical replicates, for each genotype. Data was analyzed using built-in FORMAX 2.7 software, using *Solanum lycopersicum* (Linnaeus, 1753) and *Glycine max* (Linnaeus, 1753) Merril, 1917 genomes as size standards, according to Doležel et al. (1992).

### Metaphase spreads

Meristem cells from root tips were isolated to obtain metaphase chromosome spreads. Root tips were collected from at least five different plants, of each genotype, then fixed in ethanol: glacial acetic acid (3:1v/v) solution for 60 min at 4 °C and finally digested with 2 % cellulase and 20 % pectinase (Maluszynska and Heslop-Harrison 1993, Schwarzacher and Heslop-Harrison 2000). Each root tip was squashed in a drop of 60 % acetic acid on a histological slide, under a cover glass. The cover glass was then removed using liquid N_2_ and the slide, air-dried. Slides containing chromosomes with high quality were selected using phase contrast mode in the AxiosKop microscope (Zeiss, Oberkochen, Germany).

### DAPI staining

Slides containing metaphase spreads were stained with DAPI (4’, 6-diamino-2-phenylindole; 2 µg/ml) to determine the presence of heterochromatic bands (AT-rich regions). The chromosomes were analyzed using the epifluorescent Zeiss AxioPhot photomicroscope (Zeiss, Oberkochen, Germany), with the corresponding DAPI fluorescent filter. Images were captured using the Zeiss AxioCam MRc digital camera (Carl Zeiss Light Microscopy, Göttingen, Germany) and Axiovision Rel. 4.8 software (https://www.zeiss.com/microscopy/int/products/microscope-software/axiovision.html). Images were acquired and further analyzed using the Adobe Photoshop CS software, applying only functions, except cropping, that affect the whole image equally.

### *CMA_3_* banding

For CMA_3_ banding, the nuclear dye chromomycin A3 (CMA_3_, Sigma Aldrich) was used following Schweizer and Ambros (1994). Aged slides (72 h) were treated with CMA_3_ and the slides mounted with glycerol / McIlvaine buffer, added to MgCl_2_. Slides were observed in the Zeiss AxioPhot photomicroscope, with the CMA_3_ corresponding fluorescent filter. Capture and treatment of the images were performed as described above.

### GISH

Genomic DNA from all four genotypes was isolated according to the CTAB protocol (Ferreira and Grattapaglia 1998) in order to obtain the probes for GISH. Four young leaflets, collected from five different plants, for each genotype were assembled to form three DNA pools, for each genotype. Purified DNA (1µg) was then labeled with, either digoxigenin-11-dUTP (Roche Diagnostics Deutschland GmbH) or Cy3-dUTP (Roche Diagnostics Deutschland GmbH) by Nick Translation (Roche Diagnostics Deutschland GmbH). Incorporation of digoxigenin labeled nucleotides and the estimate concentration of the probes were determined by dot blot, followed by immunocytochemical detection. Metaphase spreads were pre-treated with RNase A and pepsin prior to fixation with 4 % paraformaldehyde and then with the hybridization solution, as described by Schwarzacher and Heslop-Harrison (2000).

GISH was performed according to Schwarzacher and Heslop-Harrison (2000). To obtain the *A. hypogaea* probe, approximately 50 ng/µl/slide of the genomic DNA of *A. hypogaea* was used. Similar amount of IpaDur1 genomic DNA was used to prepare the other probe. Hybridizations were carried out for 16 h at 37 °C, followed by 73 % stringent washes.

For single GISH, metaphase spreads of IpaDur1were hybridized with the *A. hypogaea* probe. After analysis and images acquisition, the *A. hypogaea* probe and DAPI stain were removed (Heslop-Harrison et al. 1992), and the same slides were re-hybridized with the IpaDur1 probe and DAPI. On the same way, the *A. hypogaea* chromosomes were hybridized with the IpaDur1 probe and then, with its own probe. No blocking DNA (unlabeled DNA) was used. The hybridization sites were detected using the antibody anti-digoxigenin conjugated to fluorescein (Fab fragments from sheep; Roche Diagnostics Deutschland GmbH) or by the direct observation of the Cy3 fluorescence. Chromosomes were counterstained with DAPI after the hybridization detection step in the case of digoxigenin labeled probe or after stringent washes whenever the probe was labeled with Cy3. Images were captured using corresponding fluorescent filters for DAPI, FITC and Cy3 and the analyses conducted as described before.

For double GISH, approximately 50 ng/µl/slide of each diploid labeled DNA was used concomitantly. Slides were hybridized as above described, with no blocking DNA. Detection of hybridization sites, DAPI counterstaining, analysis and images acquisition were conducted as described above.

### 5S and 45S rDNA chromosome mapping

The ribosomal sequences (rDNA) coding for 5S and 45S (18S-5.8S-25S) of *Lotus japonicus* (Regel) K. Larsen, 1955 (Pedrosa et al. 2002) and *Arabidopsis thaliana* (Linnaeus, 1753) Heynhold, 1842 (Wanzenböck et al. 1997), respectively were used to obtain the rDNA probes for FISH. DNA was labeled with either digoxigenin-11-dUTP or Cy3-dUTP by Nick Translation (Roche Diagnostics Deutschland GmbH).

### *LTR retrotransposons chromosome mapping*

The LTR retrotransposon families, RE128-84 (Genbank KF729744.1; KF729735.1; KC608796.1; KC608788.1), representing the Ty1-copia group; Pipoka (Genbank KF729742.1 and KC608774.1) from Ty3-gypsy and Athena (Genbank KC608817.1), a non-autonomous transposon (which lacks the reverse transcriptase coding sequence) were chosen as the representatives of the most abundant LTR-retrotransposon families, and amongst the most and least frequent LTR-retrotransposons in *A. duranensis* and *A. ipaensis* genomes. DNA corresponding to the sequence coding for the reverse transcriptase enzyme of RE128-84 (Revtrans-RE) and Pipoka (Revtrans-PIP) were used to obtain the probes for FISH. Since Athena family comprises non-autonomous elements, there is no DNA sequence coding for the reverse transcriptase enzyme. Therefore, a non-genic, internal conserved DNA sequence, specific to the Athena family (Conserved-Ath) was used to obtain Athena probe. DNAs were PCR-amplified and the size of the amplicons confirmed in 1 % (w/v) agarose gel. DNAs were then purified and sequenced. Each DNA was labeled with either digoxigenin-11-dUTP or Cy3-dUTP by Nick Translation (Roche Diagnostics Deutschland GmbH). Primers, sizes of the amplicons and the sequences are listed in Table 2. Hybridization conditions, detection of the hybridization sites, DAPI counterstaining, analysis and images acquisition were conducted as described above.

**Table 2.** Characteristics of the LTR-retrotransposon families, RE128-84; Pipoka and Athena. Conserved DNA sequence used as probes; transposition autonomy character; superfamily; primers for amplification; sequences, sizes and names of the amplified DNA.

**RT-LTR**	**Superfamily**	**Primers**	**Name and fragment size (bp)**	**DNA conserved sequences**
**Athena non-autonomous**	-	Athena-FWD CCATCATAATTATCATAGTTGTGG Athena-REV CTCCAAACCAAGAGGGTGATAAC	Conserved-Ath 618	TTATGGAAAGGAAGGGATCCCATAACTCATCCCAAGTCAAGGTTTCATTACGTTTTAAACCACTTTTTCATCAATTTTGAGTCTTACTTGTTTATATTAGATACATAGTTCTTTTATTCCTTCATTAGTTTATTAATTACAATTTTGCCTTGTTCTTTTATCTCTTTATTGTTTACTTCAAACATTGAAAACCCTTTTGATCTTCACAACCAATTTTATGCACTTGTTGTCACTAGTTCCTAGGGAGAACAAATACTCTCGGTATATATATTTGCTTTGAATTGTGACAATCTTTAGAGTAATAATTTGACTATTGGCCAATTGTTGGTTCGAAGCTATACTTGCAACGAAGATCTATTTGGAGAAAATTCCAACCTACAATTTGGTCTTTGTCAAATTTTGGCGCCGTTGCCGGGGAGCTAATGTCATGAGTGCTATATTTTGGTTGTTGTAAATATGTCCATAGTATGAATAGATACTTTTTGGTTGCTTGTTTATTTTTGTTGGTAATTAGGATTTTGTTTATTTTGTTAATTGATGTCTTTAGTTGTTATTTTCAATTTTCTCTATGA
**RE128-84 autonomous**	Ty1-*Copia*	RE128-84-FWD CCACTAGATCCTCAAGCAAG RE128-84-REV AGAAGGCACTAAGCCTTTC	Revtrans-RE 558	AGCAAGAAGCAAGTAGAACCGAGCAATGTTGCCTTCTTGTCCCAATTGGAGCCTCTCAATGTGAAACAAGATCTTGAAGACCCCTCATGGGTTAAAGCCATGGAAAAAGAGCTGGCACAATTTGAAAAGAATGAGGTGTGGACACTTGTACCAAATCCAAATGATAAGAAGGTAACCGGTACAAGGTGGATTTTTAAAAATAAATTGGTTGAGGATGGTAGTGTTGTTCGTAACAAGGCTAGATTAATGGCCCAAGGTTACGATCAAGAAGAAGGAATTGATTTTGATGAGTCATTTTCCCCGGTAGCTAGAATGGAAGCAATTAGGTTGCTTCTTGCCTATGCTGCCCACAAGGGTTTTCAAGATGTTCCAAATGGATGTCAGATGTGCATTCCTTAATGGTTTTATAGATAGGGAAGTATTTGTGACTCAACCCCTCGGTTTTGAAAGTAAAGAATTTCCAAACCATGTTTTTAAATTATCAAAGGCTCTTTATGGCCTTAGGCAAGCTCCAAGAGCTCGGTAT
**Pipoka autonomous**	Ty3-*Gypsy*	Pipoka-FWD CCACATTGCTTTAGAGGATC Pipoka-REV GCTTGTCAAAAGCCTCCATGC	Revtrans-Pip 535	AAGAAAAAACAACCTTTACATGCCCCTTTGGCACTTATGCCTACAAGCGTATGCCATTTGGCTTATGCAACGCACCGGTAACTTTCCAAAGGTGTATGATGAGCATATTTGCAGATCTTCAAGAGCATTGGATGGAGGTGTTCATGGACGATTTTAGTGTCTATGGGGACTCTTTTGATCTTTGCTTGGACAACCTTGCAAAAGTGTTGGAGAGGTGTACTAAAACAAATATTGTCTTAAATTTTGAGAAGTGTCATTTTATGGTTAGACAAGGTATTGTTTTAGGACACATTATCTCTAACGATGGTATTTCTATGGATCCAGCAAAGATAAATGTTATATCTAGTTTACCTTACCCCTCCTCCGAGAGGGAAGTCCGTGCGTTCCTTGGACATACAGGTTTTTACTGGTGATTTATTAAGGACTTTAGCAAGGTGGCATTACCTCTATCTTGATTGTTGCAAAAAGACGTTGAATTTGATCGAAGCAAAGAGT

### In silico coverage and mapping of the LTR-retrotransposons on the diploid genomes

The conserved DNA sequences specific for each LTR-retrotransposon family (Table 2) were used as the query to assess the estimate coverage of each LTR-retrotransposon in *A. duranensis* and *A. ipaensis* diploid genomes, using the REPEATMASKER (www.repeatmasker.org), with default parameters, except with the parameters -nolow and -norna to not mask low-complexity sequences and rDNA. The estimate coverage included all members of each LTR-retrotransposon family, thus including complete sequences, reminiscent fragments, nested sequences and solo LTRs. Output files were processed using a custom Perl script, and regions masked by more than one sequence in the repeat library were recognized and counted only once.

These conserved DNA sequences from each LTR-retrotransposon family were used as queries to assess their distribution in the chromosomal pseudomolecules, of both diploid species, using the PeanutBase BLAT tool (http://www.peanutbase.org). The match score was set to ≥ 80 %. Data was manually curated to remove sequences with different size than the expected one (Table 2); misalignments, overlapping of similar sequences and tandem organized sequences, here considered as a single hit. After trimming, the number of hits for each LTR-retrotransposon was determined for each chromosomal pseudomolecule, designated Aradu.A01 or Araip.B01, for example, according to nomenclature previously used (Bertioli et al. 2016, http://www.peanutbase.org). To avoid confusion of cytogenetic and pseudomolecule numbering, which might not fully correspond, here in this manuscript, the cytogenetic numbering will be prefix with “cyt- xxx” (for example, cyt-A1, for chromosome 1 of the subgenome A and cyt-B1 for chromosome 1 of the subgenome B), for both allotetraploids and diploids.

## Results

### Genome sizes

The DNA content estimated by flow cytometry revealed that IpaDur1 had a value very close to the sum of those of *A. duranensis* and *A. ipaensis*, however, slightly different from that of *A. hypogaea* (Table 1). Therefore, the estimate size of IpaDur1 genome is 2.89 Gb. The DNA content of the accession V14167 of *A. duranensis* was herein determined for the first time and its value was very close to those previously determined for other accessions of this species (Temsch and Greilhuber 2001, Samoluk et al. 2015a, b). On the other hand, the herein estimate value for *A. ipaensis* (3.34 pg) was slight higher than previous data (3.19 pg; Samoluk et al. 2015a, b).

### Organization of chromosomes

**Figure 1.**
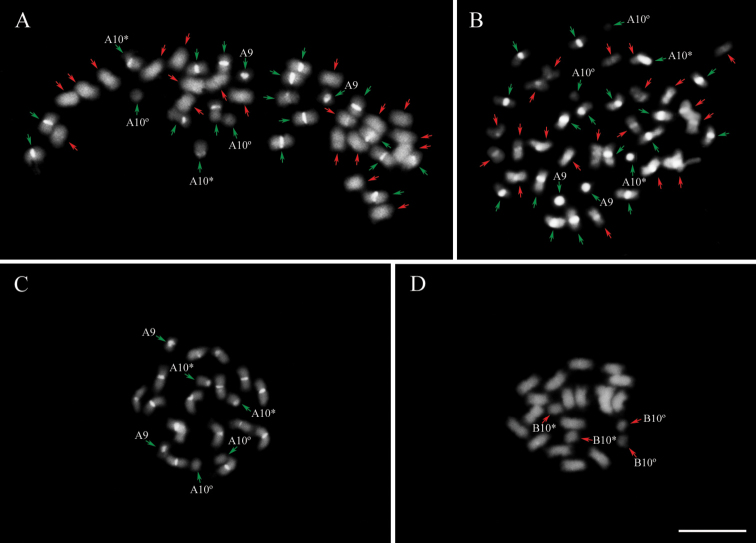
Metaphase chromosome spreads from root tips after DAPI staining (bright white) of **A** IpaDur1 **B** *A. hypogaea* **C** *A. duranensis* and **D** *A. ipaensis*. Chromosomes of the A subgenome (green arrows) and B subgenome (red arrows). Cyt-A9 (A9). Whenever the secondary constriction on cyt-A10 and cyt-B10 is extended, forming the thread-like constriction; the short arm and the proximal segment of the long arm are indicated by an asterisk (*) and the separated satellite is marked by a degree sign (°). Bar = 5μm.

**Figure 2.**
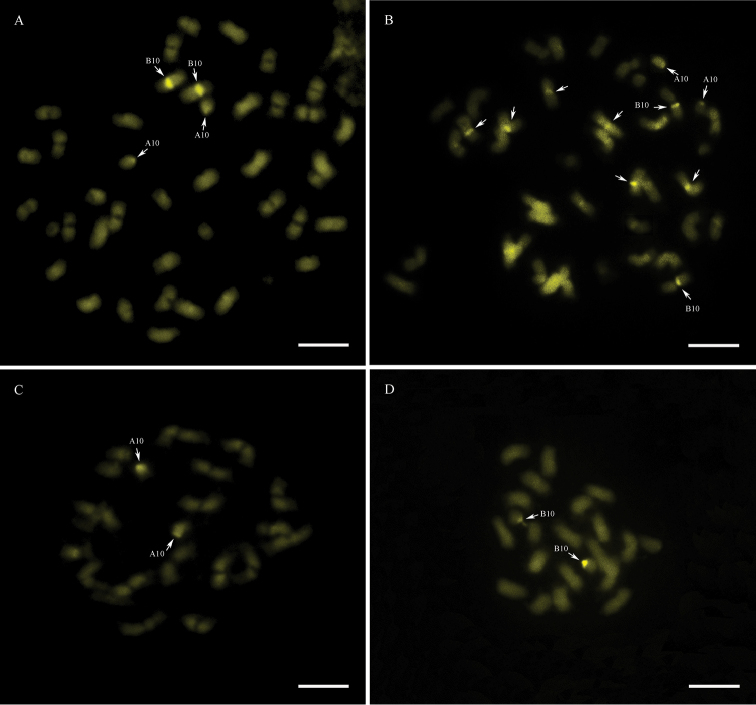
Chromosomes with CMA_3_^+^ bands (arrows) on their proximal regions. **A** IpaDur1 **B** *A. hypogaea* **C** *A. duranensis* **D** *A. ipaensis*. Cyt-A10 (A10) and cyt-B10 (B10). Bar = 5μm.

IpaDur1 harbored 40 chromosomes, with similar morphology to those chromosomes of *A. hypogaea* and their progenitors, *A. ipaensis* and *A. duranensis*, being mostly metacentric (36 m + 4 sm), with the two submetacentric pairs of chromosomes designated as cyt-A10 and cyt-B10, both SAT chromosomes (Table 1; Fig. 1A). IpaDur1 A subgenome chromosomes, as well as those of *A. hypogaea* and *A. duranensis* had evident DAPI^+^ bands, situated at centromeric regions (Fig. 1A, B, C). DAPI^+^ bands on B subgenome chromosomes of both allotetraploids, as well as those on the chromosomes of *A. ipaensis* were not detected (Fig. 1D). Proximally located CMA_3_^+^ bands (DNA regions rich in C-G) on cyt-A10 and cyt-B10 were observed in the four genotypes analyzed (Fig. 2A, B, C, D). However, *A. hypogaea* had another three pairs of chromosomes with CMA_3_^+^ bands (Fig. 2B). The karyotype formulae and CMA_3_^+^ banding patterns are compiled in Table 1.

### GISH

**GISH with the allotetraploid genomic probes**

**Figure 3.**
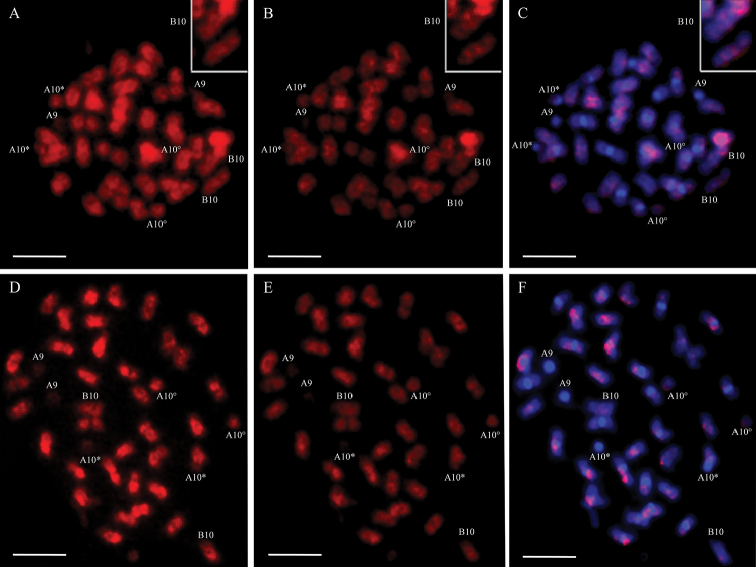
Single GISH on IpaDur1 (**A, B, C**) and *A. hypogaea* (**D, E, F**) chromosomes, followed by DAPI counterstaining (blue **C, F**). Hybridization with the genomic probe of IpaDur1 **A, E** *A. hypogaea* probe **B, D** and **C** overlapping of DAPI and *A. hypogaea* probe on IpaDur1 chromosomes **F** overlapping of DAPI and IpaDur1 probe on *A. hypogaea*. Cyt-A9 (A9), CytB-10 (B10). Insets of cyt-B10 of IpaDur1 (**A, B, C**) showing alternate dark and light bands. When the secondary constriction on cyt-A10 is extended, forming the thread-like constriction, the short arm and the proximal segment of the long arm are indicated by an asterisk (*) and the separated satellite is marked by a degree sign (°). Bar = 5μm.

Genomic in situ hybridization used either *A. hypogaea* or IpaDur1 labeled genomic DNA as the probe (single GISH). Hybridization with IpaDur1 or *A. hypogaea* probes indicated a similar and overall affinity of both probes to all chromosomes of IpaDur1, except for the signals on cyt-A9 (equivalent to Aradu.A08; Bertioli et al. 2016), which were observed only after hybridization with the IpaDur1 probe (Fig. 3A, B), but not after hybridization with the *A. hypogaea* probe. Additionally, hybridization on IpaDur1 cyt-B10 chromosomes (Fig. 3A, inset) with the IpaDur1 probe generated signals evenly distributed, all along the chromosomes, whilst signals after the hybridization with *A. hypogaea* probe had an alternated pattern, with dark and lighter bands (Fig. 3B, C, insets), indicating different affinity of this probe to different regions of these chromosomes. On the other hand, the cyt-B10 of *A. hypogaea* had signals evenly spread along the chromosomes, independently of the probe used. Furthermore, cyt-A9 of *A. hypogaea* showed weak signals, independently of the probe used (Fig. 3D, E, F), whilst in IpaDur1, the signals were evident.

**GISH with the diploid genomic probes**

**Figure 4.**
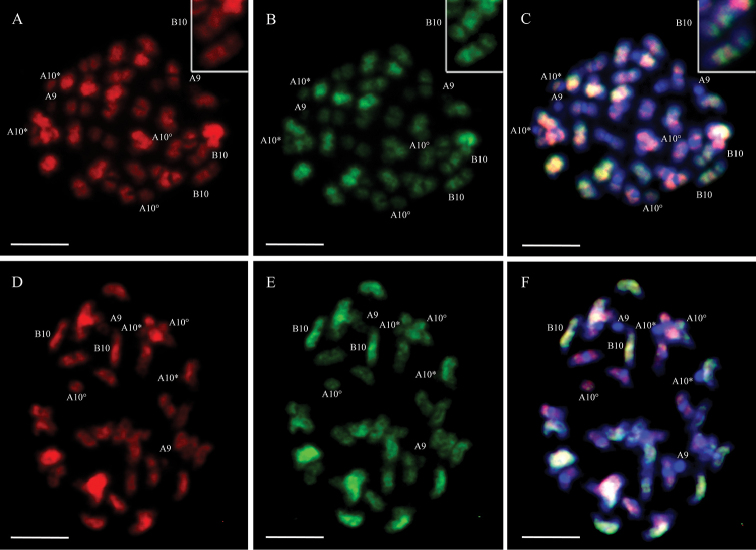
Double GISH on IpaDur1 (**A, B, C**) and *A. hypogaea* (**D, E, F**) chromosomes, followed by DAPI counterstaining (blue **C, F**). Hybridization with the genomic probe of *A. duranensis* (red **A, D**) and *A. ipaensis* (green **B, E**). Overlapping of DAPI and both diploid probes on **C** IpaDur1 and on **F** *A. hypogaea*. Cyt-A9 (A9), cyt-B10 (B10). Insets of IpaDur1 cyt-B10 (**A, B, C**), showing a colored mosaic. When the secondary constriction on cyt-A10 is extended, forming the thread-like constriction, the short arm and the proximal segment of the long arm are indicated by an asterisk (*) and the separated satellite is marked by a degree sign (°). Bar = 5μm.

Simultaneous hybridization with *A. duranensis* and *A. ipaensis* genomic probes (double GISH) confirmed that each diploid probe hybridized preferentially with the chromosomes of its corresponding subgenome, for both IpaDur1 and *A. hypogaea*. IpaDur1 showed evident hybridization on all chromosomes, as single or overlapping signals (one or both probes hybridizing to the same region of the chromosome, respectively), except for cyt-A9; centromeres of A subgenome chromosomes and terminal chromosomal regions, which hybridized poorly (Fig. 4A, B, C, D).

Strikingly, a distinct intercalated mosaic-banding pattern was also observed on the pair of chromosomes cyt-B10: bands with higher affinity to *A. duranensis* genomic probe (Fig. 4A, inset) and bands with higher affinity to *A. ipaensis* probe (Fig. 4B, inset). This pattern on IpaDur1 cyt-B10 is similar to that observed after single GISH using the *A. hypogaea* probe (intercalated dark and light bands) (Fig. 3B, C). These lighter bands correspond to the subgenome A of IpaDur1, as showed after double GISH, which showed stronger signals with *A. duranensis* probe, whilst the dark bands correspond to the B subgenome, as indicated after hybridization with *A. ipaensis* probe. Together, results suggest that the A subgenome component in the cyt-B10 of IpaDur1 might had changed after polyploidization, or that it is derived from a different accession of *A. duranensis*. At least another chromosome pair of IpaDur1 chromosomes also appears to show weaker affinity to the *A. duranensis* probe in one part, and stronger affinity to *A. ipaensis* probe, in another.

*A. hypogaea* chromosomes showed patterns similar to those observed in IpaDur1 after double GISH, except cyt-B10 that showed uniform hybridization signals along the chromosomes (Fig. 4D, E, F). Both allotetraploids had few signals on centromeres of the A subgenome chromosomes and terminal regions of all chromosomes, after both (single and double) GISH. These results are compiled in Table 1.

### 5S and 45S rDNA chromosome mapping

**Figure 5.**
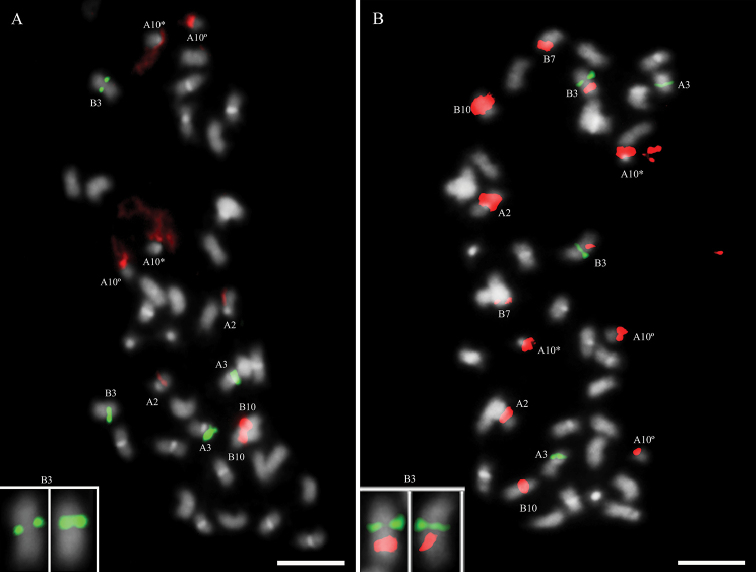
**A** IpaDur1 and **B** *A. hypogaea* chromosomes hybridized with the 5S rDNA probe (green) and 45S (red), followed by DAPI counterstaining (bright white). Cyt-A2 (A2), cyt-A3 (A3), cyt-B3 (B3), cyt-B7 (B7) and cyt-B10 (B10). *A. hypogaea* cyt-B3 with the co-localization of 5S and 45S rDNA signals. When the secondary constriction on cyt-A10 is extended, forming the thread-like constriction, the short arm and the proximal segment of the long arm are indicated by an asterisk (*) and the separated satellite is marked by a degree sign (°). Bar = 5μm.

**Figure 6.**
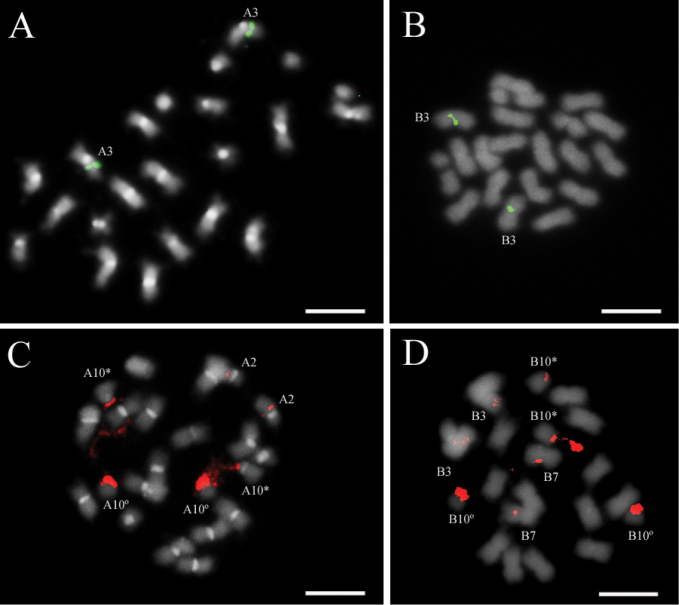
Mitotic metaphase chromosome hybridized with the 5S rDNA probe (green **A, B**) and 45S rDNA probe (red **C, D**), followed by DAPI counterstaining (bright white). **A** *A. duranensis* (2n = 2x = 20) showing signals on cyt-A3 (A3) **B** *A. ipaensis* (2n = 2x = 20) showing signals on cyt-B3 (B3) **C** *A. duranensis* with signals on cyt-A2 (A2) and cyt-A10 (A10) **D** *A. ipaensis* showing signals on cyt-B3, B7 and B10. When the secondary constriction on chromosome 10 is extended, forming the thread-like constriction, the short arm and the proximal segment of the long arm are indicated by an asterisk (*) and separated satellite is marked by a degree sign (°). Bar = 5μm.

The number of 5S rDNA loci was an additive character for both IpaDur1 and *A. hypogaea*: one locus on the cyt-A3, originating from the corresponding chromosome in *A. duranensis*, and another locus on cyt-B3, from the corresponding chromosome in *A. ipaensis* (Fig. 5A, B). Observations of cyt-A3 in both IpaDur1 and *A. hypogaea* indicated that the 5S signals extended from the proximal into the interstitial chromosomal regions (Fig. 5A, B), whereas in the corresponding chromosomes of *A. duranensis*, the signals were restricted to the proximal region (Fig. 6A). Further analysis on meiotic chromosomes is needed to confirm the possible increase of these loci in allotetraploids. The 5S rDNA signals on cyt-B3 had a similar pattern in both allotetraploids and *A. ipaensis* (Fig. 6B).

Considering the FISH with the 45S rDNA probe, there were only three loci in IpaDur1 and five *A. hypogaea*, thus being an addictive character only for the latter. In IpaDur1 (Fig. 5A), the signals were proximally located on cyt-A2 and cytB-10, while on cyt-A10, signal was near the secondary constriction of the SAT region, forming a thread-like constriction, characteristic of NORs (Nucleolar Organizing Regions), as observed on the corresponding chromosome of *A. duranensis* (Fig. 6C). The *A. hypogaea* 45S rDNA loci (Fig. 5B) were proximally located on cyt-A2, cyt-B10 and cyt-B3; terminally positioned on cyt-B7 and, on cyt-A10, they were situated near the secondary constriction of the SAT region, as observed in the corresponding chromosome of the progenitor diploid species, *A. duranensis* (Figs 5B; 6C, D). No differences were detected in the signals produced by either 5S and 45S rDNA dig-dUTP or Cy3-dUTP labeled probes. Interestingly to note that the co-localization of 5S and 45S rDNA loci on cyt-B3 was detected only in *A. hypogaea*, but not in IpaDur1 (Fig. 5A, B, inset). In contrast, the 45S rDNA loci co-localized with CMA_3_^+^ bands on cyt-A10 and cyt-B10, for both allotetraploids, as well as on the corresponding chromosomes, for both diploid species. FISH results are summarized in Table 1.

### LTR-retrotransposon chromosome mapping

**RE128-84**

**Figure 7.**
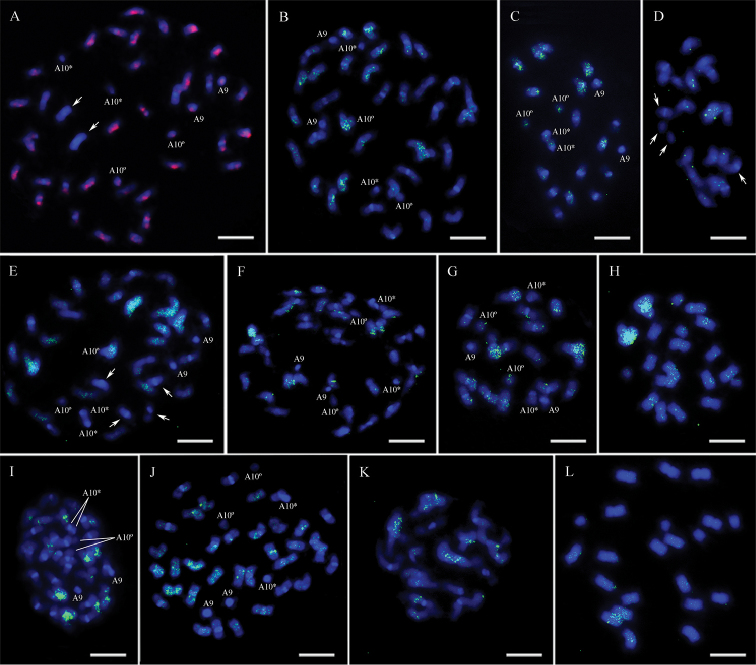
IpaDur1 (**A, E, I**), *A. hypogaea* (**B, F, J**), *A. duranensis* (**C, G, K**) and *A. ipaensis* (**D, H, L**) chromosomes hybridized with the LTR-retrotransposon probes RE-128-84 (**A, B, C, D**), Pipoka (**E, F, G, H**) and Athena (**I, J, K, L**), followed by DAPI counterstaining (blue). Cyt-A9 (A9). Chromosomes lacking signals (arrow). When the secondary constriction on cyt-A10 is extended, forming the thread-like constriction, the short arm and the proximal segment of the long arm are indicated by an asterisk (*) and the separated satellite is marked by a degree sign (°). Bar = 5μm.

In all genotypes, the RE128-84 signals were preferentially dispersed on proximal regions and along the arms of the chromosomes, and seldom detected on centromeric and terminal regions. For both allotetraploids (Fig. 7A, B), the majority of the chromosomes had signals, except on cyt-A9 and cyt-A10 of *A. hypogaea* and on another pair of A subgenome chromosomes of IpaDur1. Signals lacked also on two pairs of chromosomes in *A. ipaensis*, whilst *A. duranensis* showed overall more evident signals than those in the other diploid species (Fig. 7C, D). However, chromosomes of the subgenome B of both allotetraploids generally had more signals than on *A. ipaensis* chromosomes, although no quantitative analysis could be performed.

**Pipoka**

As for RE128-84, Pipoka signals observed were spread along the chromosomes, except on centromeric and terminal regions. The majority of the IpaDur1 chromosomes showed signals (Fig. 7E), while *A. hypogaea* had only few signals (Fig. 7F). *A. duranensis* showed comparable signals than those in *A. ipaensis*, with signals on all chromosomes of both diploid species (Fig. 7G, H). This probe did not hybridize to cyt-A9 or cyt-A10 of both allotetraploids and just poorly hybridized on these same chromosomes of *A. duranensis*. The hybridization patterns on the chromosomes of IpaDur1 suggested being closer to those detected in both diploid species than those observed for *A. hypogaea*.

**Athena**

In a similar way, chromosomes of all genotypes had *Athena* dispersed signals that lacked on centromeric and terminal regions. The abundance of signals in IpaDur1 seemed to be lower than in *A. hypogaea* (Fig. 7I, J), whilst *A. duranensis* apparently showed more signals than *A. ipaensis* (Fig. 7K, L). The signals in IpaDur1 were mostly on the B subgenome chromosomes, whilst in *A. hypogaea*, the signals were present on chromosomes of both subgenomes (Fig. 7I, J). Athena signals lacked on cyt-A9 and cyt-A10 of IpaDur1, *A. hypogaea* and *A. duranensis* (not shown). The hybridization patterns on IpaDur1 chromosomes suggested being closer to the sum of those in the diploid species, while in *A. hypogaea*, signals seemed to be more abundant. Results of the LTR- retrotransposons FISH are compiled in Table 1.

### LTR-retrotransposons coverage and mapping on pseudomolecules

The coverage of the LTR-retrotransposons indicated that these elements covered for RE128-84 family, around 1.20 % and 1.17 % of the *A. duranensis* and *A. ipaensis* chromosomal pseudomolecules, respectively, for Pipoka, 2.81 % and 6.09 % and for Athena, 0.77 % and 1.19 %. These three families covered about 4.68 % and 8.44 % of *A. duranensis* and *A. ipaensis*, mostly due to the large abundance of Pipoka members (Table 3). The *A. duranensis* pseudomolecules with the lowest and highest frequencies were respectively, Aradu.A08 and Aradu.A01, while in *A. ipaensis*, Araip.B03 and Araip.B07.

**Table 3.** In silico coverage of the LTR-retrotransposons on the chromosomal pseudomolecules of *A. duranensis* and *A. ipaensis* (accordingly to www.peanutbase.org).

Frequency of LTR-retrotransposons (%)				
Pseudomolecule	RE128-84	Pipoka	Athena	Total/ pseudomolecule
Aradu.A01 (≅ 107 Mb)	1.14	3.42	0.96	5.51
Aradu.A02 (≅ 93 Mb)	1.28	2.62	0.52	4.42
Aradu.A03 (≅ 135 Mb)	1.12	2.76	0.74	4.62
Aradu.A04 (≅ 123 Mb)	1.35	2.90	0.58	4.83
Aradu.A05 (≅ 110 Mb)	1.17	2.49	0.59	4.25
Aradu.A06 (≅ 112 Mb)	1.10	2.99	0.66	4.75
Aradu.A07 (≅ 79 Mb)	1.38	2.37	0.64	4.38
Aradu.A08 (≅ 49 Mb)	1.67	0.85	0.25	2.76
Aradu.A09 (≅ 120 Mb)	1.08	3.24	0.73	5.06
Aradu.A10 (≅ 109 Mb)	1.09	3.20	0.75	5.05
Total in A genome (1.25 Gb)	1.20	2.81	0.77	4.68
Araip.B01 (≅ 137 Mb)	1.07	6.54	1.27	8.89
Araip.B02 (≅ 108 Mb)	1.30	5.22	1.09	7.61
Araip.B03 (≅ 135 Mb)	1.21	4.75	0.97	6.93
Araip.B04 (≅ 133 Mb)	1.26	6.03	1.05	8.34
Araip.B05 (≅ 149 Mb)	1.09	6.40	1.28	8.77
Araip.B06 (≅ 137 Mb)	1.03	5.76	1.09	7.89
Araip.B07 (≅ 126 Mb)	1.09	7.61	1.32	10.01
Araip.B08 (≅ 129 Mb)	1.35	6.08	1.25	8.67
Araip.B09 (≅ 147 Mb)	1.20	5.91	1.25	8.36
Araip.B10 (≅ 136 Mb)	1.10	6.49	1.25	8.84
Total in B genome (1.56 Gb)	1.17	6.09	1.19	8.44

Mb: megabase. Gb: gigabase.

**Figure 8.**
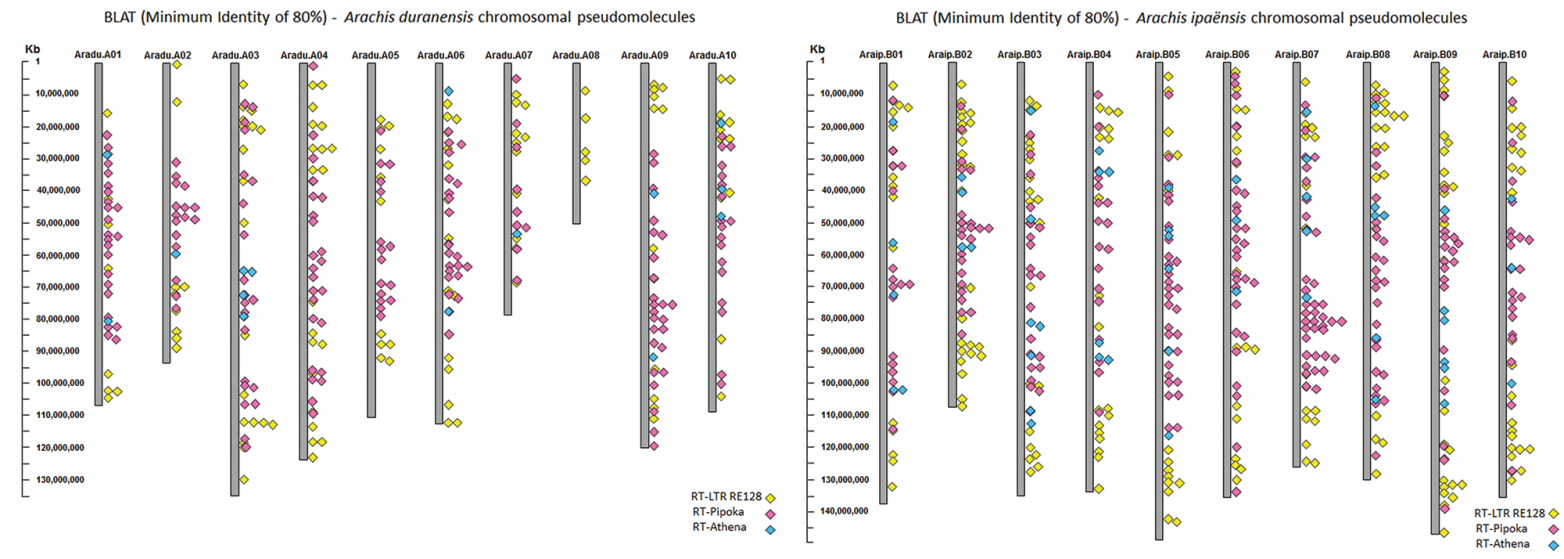
In silico mapping of the LTR-retrotransposon families, RE128-84, Pipoka and Athena on the chromosomal pseudomolecules of *A. duranensis* (left) and *A. ipaensis* (right).

Accordingly, the number of LTR-retrotransposon hits after the LTR-retrotransposons in silico mapping on the diploid pseudomolecules were higher in *A. ipaensis* than in *A. duranensis* (Fig. 8), with the pseudomolecules with the highest and lowest number of hits being in accordance with the results of the estimate coverage. RE128-84 hits were on all pseudomolecules, but more abundant on Aradu.A04 and Araip.B02. Hits were found along the arms, but not on centromeric regions. Pipoka hits were more abundant on Aradu.A09 (do not correspond to the cyt-A9) and Araip.B07; less abundant on Aradu.A07 and Araip.B01, and lacked on Aradu.A08. Hits were mostly concentrated on centromeric and proximal regions, for both diploids, and lacked on terminal regions of most of the pseudomolecules, except for Aradu.A09 and on some *A. ipaensis* pseudomolecules. Because of the low number of hits generated by Athena, no clear distribution pattern could be recognized, although the highest number of hits was on Aradu.A03 and Araip.B03; the lowest on Aradu.A02, Aradu.A07 and Araip.B10, whilst no hits were observed on Aradu.A04, Aradu.A05 and Aradu.A08 (Fig. 8).

The distribution of the LTR-retrotransposons, both in silico and in situ showed general similar patterns for the RE128-84 and Athena in *A. duranensis*; Pipoka in *A. ipaensis* and Athena, for both diploid genomes. The results shared by these two approaches enabled the inference of putative assignments by numbers for some of the IpaDur1 chromosomes, based on the abundance of hits on the numbered pseudomolecules (www.peanutbase.org). For example, *A. duranensis* chromosomal pseudomolecule Aradur.A04 had the largest number of RE128-84 hits; therefore, the chromosomes with more abundance of RE128-84 in situ hybridization signals in IpaDur1 could be putatively assigned as cyt-A4. In this same way, the pseudomolecule Araip.B02 was the one with the highest number of RE128-84 hits in *A. ipaensis*, thus the pair of chromosomes with more abundance of in situ signals would be called cyt-B2. Additionally, Araip.B07 had more Pipoka hits; therefore, the putative corresponding chromosome would be the cyt-B7. Aradur.A05 and Aradur.A08 pseudomolecules had no Athena hits, thus the corresponding chromosomes lacking in situ signals would be cyt-A5 and cyt-A9.

## Discussion

Cultivated peanut (*A. hypogaea*) is an allotetraploid with an AABB type genome, originated from the diploid progenitor wild species *A. duranensis* (A genome; female progenitor) and *A. ipaensis* (B genome; male donor) (Kochert et al. 1991, Seijo et al. 2007, Moretzsohn et al. 2013). Earlier evidence from cytogenetics, genetic mapping and analysis of progeny derived from crosses of *A. hypogaea* with an induced allotetraploid [(*A. ipaensis* K30076 × *A. duranensis* V14167)^4x^] (Fávero et al. 2006) showed that their genomes had not undergone large-scale rearrangements since polyploidization (Fávero et al. 2015, Ramos et al. 2006, Seijo et al. 2007, Foncèka et al. 2009, Shirasawa et al. 2013).

However, considering the behavior of other polyploids in general, it seemed that some changes following polyploidy were extremely likely to have occurred. Accordingly, comparisons at the genome sequence level have shown some recombination between the subgenomes of *A. hypogaea* and evidence of the A subgenome erosion by gene conversion with the B subgenome (Bertioli et al. 2016). Additionally, although meiotic pairing in *A. hypogaea* is described as presenting the bivalents, with rare univalents, trivalents, and quadrivalent exceptions (Husted 1936), there is an indication of limited homeologous pairing between A and B subgenomes, as the recent genetic studies suggested that cultivated peanut may be better classified as a segmental allotetraploid with predominantly disomic, but partially tetrasomic genetics (Leal-Bertioli et al. 2015, Bertioli et al. 2016, Clevenger et al. 2017).

In this study, in order to investigate genome structure alterations, cytogenetics was used to make a detailed comparison of *A. hypogaea*, an induced allotetraploid IpaDur1 [(*A. ipaensis* K30076 × *A. duranensis* V14167)^4x^] and their progenitor species, *A. duranensis* and *A. ipaensis*. The use of an induced allotetraploid is advantageous because this hybrid approximates an early *A. hypogaea*, and it was expected to undergo similar changes to those that peanut underwent in the early generations following polyploidy, although *A. duranensis* was the male progenitor in IpaDur1 and the female in *A. hypogaea*. Furthermore, comparisons are more accurate, because the exact diploid progenitors are known, and both have their reference genome sequences available.

### Genome sizes

The sum of the estimated genome sizes of the diploid species, herein using the flow cytometry was very similar to the one estimated for IpaDur1, but somewhat larger (4 %) than the one estimated for *A. hypogaea* (Table 1). This difference is small, but might indicate a different *A. duranensis* accession(s), as the A subgenome donor to *A. hypogaea* (Zhang et al. 2016), and / or that deletions in *A. hypogaea* subgenome A had occurred following polyploidy. Either explanation is very plausible, because *A. duranensis* is known to vary significantly in genome size (Temsch and Greilhuber 2001) and genome deletions in polyploids are known to be common (Ma and Gustafson 2006, Bento et al. 2008, Eilam et al. 2008, Petit et al. 2010). Although the estimate value for the DNA content of *A. ipaensis* herein determined slightly differed from previous data (Singh et al. 1996, Samoluk et al. 2015a, b), this new value estimates the genome size of *A. ipaensis* as being 22 % larger than that of *A. duranensis*, very similar to the size difference between their chromosomal pseudomolecules (29 %, Bertioli et al. 2016).

### Organization of chromosomes

Current analysis indicates that *A. hypogaea* and IpaDur1 share many similarities derived from the progenitor diploids, however variations relative to progenitors were also cytogenetically revealed during this study. Chromosomes of IpaDur1 are morphologically similar to those of *A. hypogaea* (Seijo et al. 2004, Robledo et al. 2009, Robledo and Seijo 2010), including the cultivar Tatu that was here studied. Staining of AT-rich heterochromatin with DAPI showed that both allotetraploids had additive patterns (Fig. 1A, B), that is, the sum of the patterns in the progenitor diploid species was equal to the patterns in the allotetraploids. However, the CG-rich regions revealed by the CMA_3_ did not have an additive pattern in *A. hypogaea*, since it that has bands on three extra pairs of chromosomes than the sum in the progenitor species (Fig. 2B). CMA_3_^+^ banding in *Arachis* species was described only for few species, which did not include species of the section Arachis (Cai et al. 1987, Pierozzi and Baroni 2014, Ortiz et al. 2017). It is difficult to interpret the significance of these differences currently, but the lack of CMA_3_^+^ bands could be a possible inaccessibility of the CMA_3_ fluorophore due to immediate structural changes in chromatin organization.

### *Genome affinity by GISH*

Double GISH using simultaneously both labeled genomic DNAs from the diploid species as probes and, the single GISH using separately each of the allotetraploid genomic labeled DNA as probe were used to study the overall affinities of the genomes, especially considering the known biases of hybridization kinetics related to DNA repetitive fractions. Our hybridizations generated patterns generally consistent with previous observations in *A. hypogaea* (Ramos et al. 2006, Seijo et al. 2007). Single GISH indicated that these allotetraploid genomes shared most of its contents, which correspond to that of the diploid progenitor genomes. Both IpaDur1and *A. hypogaea* showed scarcity of signals on cyt-A9, the small pair “A” (Fig. 3), indicating its low repetitive content and possible equivalence to Aradu.A08 (Bertioli et al. 2016). Double GISH confirmed the preferential hybridization of each diploid probe to its corresponding subgenome, thus allowing chromosomes of the A and B subgenome to be easily distinguished in IpaDur1 (Fig. 4), as it was previously recognized for the genome components of *A. hypogaea* (Raina and Mukai 1999, Seijo et al. 2007). Although the genome of IpaDur1, *A. hypogaea* and progenitor species share the majority of the DNA content, remarkable differences were detected in IpaDur1, such as the striking mosaic hybridization patterns observed on cyt-B10 (Fig. 4), suggesting that this pair of chromosomes might have undergone multiple recombination events between subgenomes. Relatively strong residual hybridization in its bands suggests partial, and not complete, subgenome replacement. At first sight, this different affinity to the genomes along cyt-B10 of IpaDur1 seems consistent with the recombination between Aradu.A04 and AraipB04 that has been reported in this induced allotetraploid (Leal-Bertioli et al. 2015). However, on closer inspection, it seems likely that this possible genome instability cytogenetically observed is in a different chromosome (cyt-B10 has a conspicuous constriction indicating the presence of a large 45S rDNA cluster, but Araip.B04 does not have any 45S rDNA sequences). Although A-B subgenomes recombinations between distal euchromatic regions of homeologous chromosomes have been shown in *A. hypogaea* (Bertioli et al. 2016), it is believed that most likely, they were not detected here because of their relatively small size and poor hybridization in the repeat-poor distal portions of the chromosomes.

### 5S and 45S rDNA chromosome mapping

Hybridizations with ribosomal DNAs (rDNAs) probes were carried out since they generate strong signals, the positions of ribosomal loci are important landmarks for cytogenetic chromosome identification and it is known that their concerted evolution drives changes following polyploidy (Grabiele et al. 2012). The number of 5S rDNA loci herein determined for IpaDur1 showed to be additive, as it was here confirmed for *A. hypogaea* cultivar Tatu (Fig. 5), in accordance to previous reports including other *A. hypogaea* cultivars (Seijo et al. 2004, Robledo et al. 2009, Robledo and Seijo 2010). However, the heteromorphic signal on cyt-A3, for both allotetraploids, relative to the corresponding chromosome in the diploid *A. duranensis*, could indicate possible genome instability in the allopolyploids. Similar heteromorphic signal was also observed for other *A. hypogaea* accessions by Seijo et al. (2004). Sequence similarity searches on *A. ipaensis* chromosomal pseudomolecules identified a single location of 5S rDNA, on Araip.B06, thus allowing its correspondence to cyt-B3. Nonetheless, similarity searches of the sequences of *A. duranensis* detected multiple rDNA locations (data not shown), making ascertained further cytogenetic - pseudomolecule correspondences, still a challenge.

Generally, 45S rDNA loci inherited from both parents often remain structurally (not necessarily functionally) intact in first generation hybrids, and ancient allopolyploids usually display uniparental inheritance and / or structural rearrangements of parental 45S rDNA (Volkov et al. 2017). Our analysis indicated that the sum of the 45S rDNA loci in the diploid species is equivalent to the number detected in *A. hypogaea* cultivar Tatu (Fig. 5B), in accordance to previous reports for other cultivars (Seijo et al. 2004, Robledo et al. 2009, Robledo and Seijo 2010). Nonetheless, and notably, the number of 45S rDNA loci in IpaDur1 differed from the sum of the progenitor diploid species, since signals on cyt-B3 and cyt-B7 were not detected (Fig. 5A), thus constituting another hint of genome instability. Nucleolar dominance was the same in both allotetraploids: NORs were present on cyt-B10 of *A. ipaensis* (Seijo et al. 2004), but might not be active on cyt-B10 of *A. hypogaea* (only on cytA-10; Seijo et al. 2004) or IpaDur1. Such alterations could be consequences of different mechanisms of heritance of these sequences, yet to be clarified in further studies.

Chromosome cyt-A10 of IpaDur1 (Fig. 5A) is the only pair comprising a potential active NOR (Nucleolus Organizer Region) in this genotype, since the thread-like constricted with 45S rDNA hybridization signals, typical of NORs are consistently present. In a similar way, our analysis of cyt-A10 in *A. hypogaea* (Fig. 5B), *A. duranensis* (Fig. 6C) and cyt-B10 in *A. ipaensis* (Fig. 6D) indicated similar patterns of 45S rDNA signals, which are in accordance with the previous reports for these diploid species and *A. hypogaea* (Seijo et al. 2004, Robledo et al. 2009, Robledo and Seijo 2010). On the other hand, cyt-B10 for both allotetraploids did not show a distended rDNA 45S signal, suggesting that this locus might have been silenced, and hence suggesting a nucleolar dominance of cyt-A10. The possible cyt-B10NOR silencing in this newly synthetized allotetraploid indicates that this possible nucleolar dominance could be a rapid event after polyploidization, besides being independent of the maternal or paternal role played by *A. duranensis* during allotetraploidization. NORs / rDNA 45S loci losses, such as those described for the allopolyploids hybrids *Tragopogon mirus* (G.B. Ownbey, 1950) and *T. miscellus* (G.B. Ownbey, 1950) (Soltis et al. 2004); *A. thaliana* and the natural *A. suecica* (Pontes et al. 2004) and the induced *Triticum* (Linnaeus, 1753) /*Aegilops* (Linnaeus, 1753) (Guo and Han 2014) are usually attributed to rapid chromosomal rearrangements after polyploidization, although longer periods are usually necessary for a selective elimination of one parental NORs / 45S rDNA. Moreover, if there are some DNA regions in the chromosomes with gaps or constrictions that have tendency to break/gap, among possible consequences, there are changes in number, position and activity of 45S rDNA sites (reviewed by Rocha et al. 2017).

### LRT-retrotransposons coverage and mapping

Differences in the repetitive content created, for example, by the activation of transposons, following polyploidy could explain why the variation of the intensity of signals on *A. hypogaea* chromosomes hybridized to its own genomic probe and IpaDur1 probe. In this regard, distribution of three retroelements from different classes was further inspected, both in situ and in silico: the Ty1-copia transposon RE128-84, the Ty3-gypsy transposon Pipoka, and the non-autonomous Athena (Fig. 8 and Table 3). FISH using the selected LTR-retroelements probes produced dispersed signals, corresponding to larger or smaller clusters of the members of these retroelement families. Generally, although there are some indications of changes, signals in the allotetraploids were additive, mostly considering the RE128-84 (Fig. 7A, B, C, D). This indicates that there has not been large-scale activation of these retrotransposon families after allopolyploidization. Nevertheless, in silico analysis of the coverage of these LTR-retroelements on the diploid pseudomolecules did not show a complete association with their in situ distribution.

Overall, in this study, whilst there are some indications that genome changes have occurred after polyploidy in *A. hypogaea*, they are quite small: possible nucleolar dominance and genome deletions, and indications of transposon activity. Whilst recombination between subgenomes has been clearly shown by the sequence analysis in *A. hypogaea* (Bertioli et al. 2016), evidences of similar genome rearrangements could not be detected in this species using the cytogenetic tools applied in this study. This could be due to the limited power of detection of genomic hybridization (GISH) in the euchromatic chromosomal regions. In contrast, IpaDur1 has clearly undergone further alterations that could be evidenced cytogenetically: lack of two 45S sites on B subgenome chromosomes, large-scale multiple recombination between subgenomes, in at least one chromosome pair, the cyt-B10. Yet, this pair of chromosome is probably different to the pair in which A-B rearrangements were genetically detected (Leal-Bertioli et al. 2015).

It seems that IpaDur1 has a more unstable genome, and had larger recombination between subgenomes than *A. hypogaea*. IpaDur1 might be undergoing, at least in part, a route of ‘autotetraploidization’ and genetic degradation, process that has been termed the “Polyploid Ratchet” (Gaeta and Pires 2010). Since A and B subgenomes of IpaDur1 and *A. hypogaea* are mostly similar, it may have been expected that they would have similar propensities to recombination between subgenomes and stability, when incorporated in an allotetraploid form. However, this does not seem to be the case, perhaps because a distinct *A. duranensis* accession was the A subgenome donor, inheritance has been stabilized to some degree though genetic changes and selection (Jenczewski and Alix 2004, Gaeta and Pires 2010) or due to the reversed male/female roles played by the diploid species. The exact extent and the basis of genetics present in *A. hypogaea*, the cultivated peanut, are questions still unanswered and will be pursued with further investigation.
